# “We Get Our Healing Through Traditional Ways”: Canadian Indigenous Women's Use of Violence Against Women Shelters, Mainstream Counseling, and Traditional Healing

**DOI:** 10.1177/10778012241230327

**Published:** 2024-02-04

**Authors:** Cindy L. Ogden, Leslie M. Tutty

**Affiliations:** 1 University of Calgary, Calgary, AB, Canada

**Keywords:** intimate partner violence, indigenous women, IPV counseling, VAW shelters

## Abstract

We know little about what services are accessed by Indigenous women abused by intimate partners (IPV). This mixed-methods secondary analysis examines the demographics and narratives of 40 Canadian Indigenous women regarding their use of violence against women (VAW) emergency shelters (55%), second-stage VAW shelters (7.5%), mainstream community counseling (70%), and Indigenous healing practices (42.5%). Five women who identified as LGBTQ or two-spirit accessed community services but not VAW shelters. The women had experienced severe IPV, but scored below clinical cut-offs for depression, psychological distress, and PTSD. They described strengths, concerns, and barriers in accessing services. Implications for counselors are presented.

## Introduction

In Canada, Indigenous women are abused by their intimate partners at a rate three times higher than non-Indigenous women (21% of Indigenous women compared to 7% of non-Indigenous women) (Brownridge, 2008; Brzozowski et al., 2008). In addition, Indigenous women are at greater risk of being sexually assaulted by their partners (Brennan, 2011; Brownridge, 2008) and are more likely to have experienced severe and potentially life-threatening forms of violence used against them (Brownridge, 2008). They are eight times more likely to have been murdered by intimate partners (Brownridge, 2008; Brzozowski et al., 2008).

Several authors have speculated about what factors are placing Indigenous women at greater risk for IPV. Most agree that the legacy of colonization is central, by disrupting traditional values and culture, the dominant culture's use of residential schools to breakdown family life, spiritual beliefs, and languages, as well as systemic discrimination, and racism (Andersson et al., 2010; Baskin, 2012; Olsen Harper, 2006; Puchala et al., 2010).

The destabilization of Indigenous families persists (Blackstock et al., 2004; Olsen Harper, 2006; Puchala et al., 2010; Shepard et al., 2006). Indigenous children are overrepresented in the child welfare system. While 5% of Canadian children are Indigenous, Indigenous children represent 22% of substantiated child maltreatment cases and were 12 times more likely to be placed in foster care than non-Indigenous children (Trocmé et al., 2010).

When one examines the child abuse statistics and the prevalence of IPV against Indigenous women (21%), it is clear that many Indigenous women have experienced traumatic events in their lifetimes (Hoffart & Jones, 2018). This victimization adversely affects health and increases the likelihood of traumatic reactions, including posttraumatic stress disorder (PTSD) (Collin-Vezina et al., 2009; Finkelhor et al., 2007; Söchting et al., 2007). Multiple victimizations increase vulnerability to PTSD (Jones et al., 2001; Finkelhor et al., 2007).

### Services to Address IPV

#### VAW Shelters

Women abused by intimate partners often seek safety and assistance for themselves and their children in violence against women (VAW) shelters, in the aftermath of assaults, but also after having left their partners. Across Canada, in 2010, 287 VAW transition shelters (allowing stays from 1 day to 11 weeks) and 73 women's emergency shelters (allowing stays from 1 to 21 days) were documented (Burczycka & Cotter, 2011). VAW shelters offer many services, including information about IPV and helping women access housing, jobs and schooling for their children (Hughes, 2020). The crisis counseling offered in shelters, either individually or in groups, is considered vital to assisting women fleeing abusive partners (Sullivan, 2018; Tutty, 2017).

Research on Canadian shelters consistently identifes them as important resources in providing safety to women and children (Hughes, 2020; Tutty, 2015; Wathen et al., 2015). However, concerns have been noted, such as that some staff members were too busy with administrative tasks leaving them unavailable for emotional support (Tutty, 2015). No research specific to Indigenous women's experiences in mainstream VAW shelters was found although some Indigenous women have raised concerns about racism from some staff (Baskin, 2012; Tutty, 2015).

Second-stage VAW shelters provide women with a transitional step between the short-term measures of an emergency VAW shelter and living independently. Generally, women live with their children in their own apartments; but the units have enhanced security measures to address the families’ safety needs as well as programs, services and/or supports (Tutty et al., 2008). Unfortunately, though, second-stage shelters are not commonly available. Across the prairie provinces, only 3% of the shelter admissions in Manitoba were to second-stage housing, 4% in Saskatchewan, and 5% in Alberta (Beattie & Hutchins, 2015).

#### Mainstream Counseling Approaches to IPV

There are numerous approaches to counseling for women abused by intimate partners, many of which are individual approaches (Trabold et al., 2018; Tutty, 2023). Group counseling, whether as support or therapy, is a common approach. Several strengths associated with group interventions include that women gain support from other women, see that their responses are normal and provide women the opportunity to talk about how they resisted their partner's abusive behavior, and how they protected their children (Allen et al., 2021; Tutty, 2022; Wade, 1995).

Group counseling has the potential to be meaningful for Indigenous women since, “recounting one's own story of trauma safely and with some healing benefit requires a receptive audience that bears witness” (Kirmayer et al., 2009, p. 455). However, if the group is based on the Western paradigm of individualism and self-determination (McCormick, 2009), the worldviews of many Indigenous are women ignored or dismissed.

### Indigenous Healing Approaches

Indigenous healing approaches include consultation with Elders, participating in ceremonies, gaining balance through the medicine wheel, sharing or talking circles, and healing circles. In Indigenous societies, people seek out “Elders for cultural teachings, advice, prayer, healing, and guidance” (Bird, 2007; Lavallée & Poole, 2010, 2009; Puchala et al., 2010; Salée et al., 2006). Elders conduct rituals such as pipe ceremonies, spirit dances, and sweat lodges (McCormick, 2009). They use tobacco, sage, or sweetgrass to help individuals uphold and deepen their interconnections with the world (Lavallée & Poole, 2010; Kirmayer et al., 2009).

In summary, while mainstream agencies such as VAW emergency and second-stage shelters and IPV-specific counseling are available, there is a paucity of research on the extent to which Indigenous Canadian women deem them useful and/or whether Indigenous women use traditional healing approaches. With access to a unique set of 40 in-depth interviews with Indigenous women from Canada's three prairie provinces, the current study focuses on these important questions.

## Methods

The purpose of this mixed methods secondary analysis was to understand the Indigenous women's usage and perceptions of formal support systems from Canada's prairie provinces. The original research, entitled “The Healing Journey,” was a longitudinal, mixed methods study of 658 abused women from Alberta, Saskatchewan, and Manitoba. Data were collected in seven waves between 2005 and 2009, with the ethical protocols approved by the six universities involved (Universities of Manitoba, Regina, Brandon, Calgary, Lethbridge, Winnipeg). The original longitudinal study explored “abuse experiences, health, mental health, well-being, and utilization of resources” (Tutty et al., 2021). Women were recruited from IPV agencies across the three provinces. The inclusion criteria were women: (1) 18 years of age or older; (2) had experienced IPV in the previous five years or more recently; (3) not suffering a major mental health problem that would impair comprehension or memory; and (4) are not in crisis.

Mixed methods studies include both quantitative and qualitative components (Bergman, 2011; Doyle et al., 2016). The quantitative component consisted of in-depth surveys and self-report measures to assess health, mental health, and wellbeing. Trained interviewers conducted the surveys with participants every six months over 3½ years.

Qualitative semi-structured interviews were designed by a subset of Healing Journey team members, allowing the women to provide context to their experiences of IPV; 91 women (31 women from Manitoba, 31 from Saskatchewan, 30 from Alberta) were interviewed. When available, the research assistant who administered the surveys also administered the qualitative interviews. Each participant was asked where they thought their journey/story of IPV began, where that journey/story is today, and where it is taking them in future. Probes were created for women who may be marginalized by the dominant society (i.e., Indigenous, LGBTQ, women with disabilities). The qualitative interviews were conducted about halfway through the longitudinal study, with a subset of the women from the Healing Journey. The interviews took approximately 2 to 2½ hours, scheduled at times and locations convenient to the participants.

### Standardized Measures

The Composite Abuse Scale (CAS) (Hegarty et al., 2005) is a self-report measure of partner violence with four subscales: Severe Combined Abuse, Emotional Abuse, Physical Abuse, and Harassment as well as a Total CAS score. The measure consists of 30 items about whether partners took certain actions (past 12 months), and the frequency of such actions in a six-point Likert scale of never (0), only once (1), several times (2), once per month (3), once per week (4), or daily (5). for a total possible score of 150 (Hegarty et al., 2005). The clinical cutoff for the entire scale is 3 to 7 (Hegarty et al., 2005). The CAS has strong criterion and construct validity, as well as internal reliability (*α* = .85); the subscales also have a Cronbach's alpha of .85 or above (Hegarty et al., 2005). Cronbach's alpha in the current study is .93.

The Symptom Checklist-10 (SCL-10) (Nguyen et al., 1983) is a short form of the SCL-90 which assesses mental health and distress. The 10 items of the SCL-10 ask participants to rate their level of distress in the past week on a Likert scale from never (0) to extremely (4) on six depression items, two somatization items and two phobic/anxiety items. Müller et al. (2010) suggested a clinical cutoff of 14.2. The measure has strong validity and reliability (Müller et al., 2010; Nguyen et al., 1983). Cronbach's alpha in the current study is .89.

The Center for Epidemiologic Studies Depression Scale 10 (CESD-10) is a 10-item scale, that measures level of depression for 10 items in the past week using a 4-point Likert scale from rarely (0) to most of the time (3) with a possible score range of 0 to 30. Higher scores indicate more depression (Andresen et al., 1994). The CESD-10 has good reliability and validity (Andresen et al., 1994). Internal consistency and test–retest reliability are good (Björgvinsson et al., 2013). Cronbach's alpha in the current study is .84. Björgvinsson et al. suggest that a cutoff of 15 has the best sensitivity and specificity.

The PTSD Checklist (PCL) (Blanchard et al., 1996) screens for PTSD with 17 items, five of which address re-experiencing symptoms, three items regarding avoidance or numbing, and two items about hyperarousal. Participants rate how much they have been bothered in the past month by any symptom on a Likert scale from not at all (0) to extremely (4). The measure has good validity and reliability with a suggested cut-off score of 44 (Blanchard et al., 1996). Cronbach's alpha in the current study is .92.

Well-being was assessed by the Quality of Life (QOL) Questionnaire (Andrews & Withey, 1976). The original 25-item scale was shortened by Sullivan and Bybee (1999) to nine items measuring satisfaction with the overall QOL (i.e., How do you feel about life as a whole?) on a 7-point scale and satisfaction with areas in life (i.e., How do you feel about yourself; your personal safety; the amount of fun and enjoyment you have?). Items are rated on a 7-point scale (1 = extremely pleased; 7 = terrible) and reverse-scored with higher scale scores indicating better QOL. Cronbach's alpha for QOL in the current study is .84.

### Qualitative Data Analysis

Qualitative secondary analysis re-uses existing interviews, providing the opportunity to “gain further insight on hard-to-reach populations or sensitive topics without further intrusion” (Irwin & Winterton, 2011, p. 3; Long-Sutehall et al., 2011). First-level coding entails word-by-word scrutiny of the narratives to identify prominent themes and subthemes (Braun & Clarke, 2006). Second-level coding examines the themes and subthemes to identify similarities, differences, and gaps using the constant comparative method (Thorne, 2000). NVivo 11 was used to manage the data throughout the analytic process.

## Results

Demographic information for the 40 women (eight in Alberta, 18 in Saskatchewan and 14 in Manitoba) is presented in Table 1.

**Table 1.** Women's Demographic Profile.

Variable	Categories	Means/frequency
Age (*N* = 40)		37.7 (*SD* = 11.5)
Sexual orientation (*N *= 40)	Heterosexual	35 (87.5%)
	Bisexual	1 (2.5%)
	Lesbian	2 (5%)
	Two-spirit	2 (5%)
Current partner relationship (*N *= 40)	No longer together	25 (62.5%)
	Together	15 (37.5%)
Partner's ethnic/racial group (*N* = 40)	Indigenous	22 (55%)
	White	18 (45%)
Partner's age (*N* = 39)		38.9 (*SD* = 11.4)
Partner's sex (*N* = 40)	Male	38 (95%)
	Female	2 (5%)
Length of relationship in years (*N* = 40)		7.74 (*SD *= 8)
Children? (*N* = 40)	Yes	34 (85%)
	No	6 (15%)
Age of oldest child (*N* = 34)	Children under 18	20 (58.8%)
	Adult children	14 (41.2%)
Total income in past year (*N* = 35)		$25,354 (*SD* = $25,390)
Highest education (*N* = 40)	Not completed HS	14 (35%)
	Completed HS or GED	8 (20%)
	Postsecondary: technical	5 (12.5%)
	Postsecondary: university	13 (32.5%)
Currently working (*N* = 40)	Full-time	13 (32.5%)
	Part-time/casual	6 (15%)
	Not working	21 (52.5%)
Where lived as a child? (*N* = 40)	Biological parents/relatives	29 (72.5%)
	Child welfare/institutions/adoption	11 (27.5%)
Child abuse history (*N* = 40)	No abuse	4 (10%)
	Sexual abuse	27 (67.5%)
	Physical abuse	25 (62.5%)
	Emotional abuse	30 (75%)
	Neglect	18 (45%)
	Exposure to IPV	28 (70%)
Mental health difficulties and/or illness (*N* = 39)	Yes	22 (56.4%)
	No	17 (43.6%)
Disability (limited activity/employment) (*N* = 39)	No	25 (64.1%)
	Yes	14 (35.9%)
Type of disability (*N *= 39)	No disability	25 (64.1%)
	Physical	5 (12.8%)
	Mental health	1 (2.6%)
	Physical and mental health	8 (20.5%)

The majority (82.5% or 33) identified as First Nations, and 17.5% (7) as Metis. Interestingly, while many would expect Indigenous women to have Indigenous partners, only a little more than half of the women's partners (22 or 55%) were Indigenous, while 18 (45%) were White. The women were an average age of 37.7 years while the partners were slightly older, an average age of 38.9 years. The women mostly (35 or 87.5%) self-identified as heterosexual, with five women (12.5%) as members of the LGBTQ (Lesbian, Gay, Bisexual, Transgender, Queer/Questioning) community or as Two-Spirit (Ristock et al., 2019). Thirty-eight of the abusive partners were male, and two were female. Thirty-four women (85%) had children (range from one to nine). Of these, 20 (58.8%) had children under age 18; the children of the other 14 (41.2%) were adults.

Twenty-five women (62.5%) no longer lived with abusive partners: three had separated, two were divorced, 11 had left common-law relationships, and nine had left dating partners. Of the slightly more than one-third (15 or 37.5%) who still resided with their abusive partners, four were married, seven lived common-law, and four were dating. The lengths of relationships with the abusive partners ranged from 6 months to 41 years (average 7.7 years).

The women's highest level of education varied; 35% (14) had not completed high school, while 45% (18) had attended postsecondary institutions. Of the 35 women who reported their income in the past year, these ranged from $451 to $100,000, with a mean of $25,354. Slightly over half of the women (21 or 52.5%) were not currently working, while 13 (32.5%) worked full-time and 6 (15%) worked casual or part-time.

Regarding their childhoods, almost three-quarters (72.6%) had resided with their nuclear families (26) or relatives (3). Of the other 11 women, seven (17.5%) were involved with the child protection system, foster care and/or the criminal justice system; one (2.5%) was adopted; and three (7.5%) had attended residential schools. Only four women had not experienced any childhood abuse, thus 90% had been abused as children. Of the 36 women who disclosed childhood maltreatment, over two-thirds (27 or 67.5%) had been sexually abused, 25 (62.5%) experienced physical abuse, 30 (75%) experienced emotional abuse, 18 (45%) were neglected and 28 (70%) women had been exposed to IPV against their mothers (with overlap across categories). While 22 women identified physical or mental health difficulties, only 14 (35%) described these as limiting their activities or employment, the defining feature for considering conditions as disabilities. With respect to the seriousness of the IPV, the women's scores on the CAS subscales (see Table 2) indicate that they had experienced considerable physical abuse, emotional abuse, and harassment, as well as Severe Combined Abuse (all above the clinical cut-off scores). The women's mean score of 11.4 (*SD *= 9.6) on the Symptom Checklist-10 (SCL-10) indicate that, on average, the respondents were not experiencing psychological distress. Further, the women's mean score of 11.5 (*SD* = 7.5) on the CESD-10, indicates that the women were not experiencing clinically significant depressive symptoms, on average. Similarly, the women's average scores on the PTSD Checklist of 27.0 (*SD *= 15.3) were not in the clinical range. Average scores on the QOL questionnaire (43.1; *SD *= 9.5) were slightly more positive than other studies of women IPV survivors (i.e., Bell et al., 2009; Varcoe et al., 2017), both of which reported average QOL scores of about 40 using the same measure.

**Table 2.** Women's Scores on Abuse and Mental Health Indices.

Scale	Mean Score
CAS Severe Combined (*N* = 40)	8.4 (*SD *= 7.7)
CAS Emotional Abuse (*N* = 40)	26.1 (*SD *= 15.0)
CAS Physical Abuse (*N* = 40)	14.5 (*SD *= 9.5)
CAS Harassment (*N* = 40)	7.8 (*SD *= 5.9)
CAS Total Score (*N* = 40)	56.7 (*SD *= 34.7)
SCL-10 Total Score (*N* = 40)	11.4 (*SD* = 9.6)
CESD Total Score (*N* = 40)	11.5 (*SD *= 7.5)
PTSD Checklist (*N* = 39)	27.0 (*SD *= 15.3)
QOL Checklist (*N* = 31)	43.1 (*SD *= 9.5)

### Accessing Formal Counseling Supports

Not all 40 Indigenous women had accessed formal support through VAW shelters or community counseling agencies. A little more than half (22 or 55%) had resided in emergency (VAW) shelters, three (7.5%) had lived in second-stage shelters, and 28 (70%) women accessed community counseling services. Neither of the women with female partners nor the two-spirit respondents accessed VAW-specific services, but all five women who identified as LGBTQ or as two-spirit accessed community counseling agencies. Finally, 17 women (42.5%) spoke of seeking traditional cultural teachings and ceremony to assist them to heal from their partners’ abusive behaviors. Notably, many women had accessed more than one service, program, counselor, Elder, and/or other Indigenous healing practices.

#### Violence Against Women Shelters

As previously stated, 22 (55%) women had resided in emergency VAW shelters. Yet, it difficult for some to decide to access shelter services. Thirty of the 40 respondents (75%) had initially debated whether to contact shelters, with one-third (10 of 30, or 33.3%) deciding against. Two of these 10 women had felt ashamed to go to a shelter. “The irony is that the [VAW shelter] was just right across from the street from where we were living. I was too ashamed to go there.” Another was unaware that shelters existed. For two women, going to shelter was not an option since they were employed in VAW shelters. One did not want her work colleagues to be aware of her partner's abusive behavior. The other stated, “I’ve worked in them, and I find them ineffective. A lot of the women were frustrated; the staff were frustrated.*”* For two other women, going to a VAW shelter was not an option because a member of their partner's family, or someone closely connected to their partner's family worked there. Of the 10 women who initially chose not to access a VAW shelter, two later changed their minds and became residents. Notably, three of the 22 shelter residents (13.7%) were initially turned away for lack of space. The 22 women had diverse experiences in VAW shelters, in part influenced by the services offered. Five (22.7%) respondents highlighted how helpful the shelters were for themselves and their children. “[The VAW shelter] was amazing. When I talk to the kids, it was their favourite place.” Four women (18.2%) noted the importance of being safeguarded, not only by the shelter's security features, but also by the services. “They had a teacher, so my children didn’t have to go out. It was really safe*.*” One respondent mentioned accessing an Elder and how meaningful it was to her to have, the “Wonderful ear of the Elder, and her wonderful input; her understanding of me and in such non-judgmental ways.”

Five women (22.7%) highlighted the support and help offered by the VAW shelter counselors, commenting, *“*They help you look at the positive side and ideas on what you can do, what's available [for] help.” And “I didn’t feel judged. They’re so good there. It really did give me hope that there was a life without [abuse]. It's really hopeful.” Yet, when four of the 22 (18.2%) women tried to speak to VAW counselors, rather than exploring or trying to understand their circumstances, the counselors simply gave them advice: “They were always telling you, ‘leave the relationship’ but that wasn’t helpful.” And *“*They were too busy. It would help if I could talk to somebody, not have them tell me, ‘You should do this.’”

One resident experienced overt racism.

They treat Indian women completely different. I made friends with one White girl. I asked if I could make a sandwich for my son, and the staff said, “No, this kitchen's closed.” Shuts the door. My friend asks and the staff said, “No problem.” I never went back.

About one-third of the women (8 of 22, of 36.4%) highlighted the support that they received from other shelter residents mentioning, “You begin to realize that you’re not the only one.” And “When you hear it from another woman who's been abused, it's so profound.”

Over three-quarters of the women (18 of 22, or 81.8%) discussed the services offered by the VAW shelters, including practical help, individual and group counseling. The practical assistance ranged broadly from no additional support beyond that provided to women in-residence, to helping women find resources including permanent housing, enrolling their children in school, and connecting women with mental health/counseling services. Seven (31.8%) commented on the practical help provided, however, more than one-third (3 of 7, or 42.9%) had stayed in shelters with no practical assistance. “They plunked us in a room; gave us a meal schedule and cleaning duties. They don’t realize how hard it is for women to get their own [place], or if they even have the strength to make the phonecall, book the appointment.”

Half of the women (11 of 22) spoke of their experiences with individual counselors. One described a VAW shelter that did not offer counseling, noting: “There was no counselling. Basically, a place to eat and sleep. I could have done that at home.” Three women (13.6%) did not find the individual counseling helpful because the counselors were telling them what to do, rather than letting the women decide what seemed best for their personal circumstances, or the respondents considered the counselors inexperienced.

Nevertheless, over one-third of the women (9 of 22, or 40.9%) highlighted positive experiences with shelter staff's individual counselling, and the opportunities it provided, commenting, for example, “It allowed me the opportunity to self-reflect.” And “My counsellor explained that women should be treated with respect instead of being knocked around and accused in jealousy. I started understanding.”

Over one-third (9 of 22, or 40.9%) had attended group counseling in the VAW shelters. One woman objected because attendance was mandatory; nor did she find group helpful. Yet, the other eight women (88.9%) found the groups useful, commenting, for example, “The counsellors were great. They really understood.” Two women emphasized the support that they received from other group members: being able to, “Talk to somebody that's been through the same thing, how they changed or got out of the relationship.” These respondents believed that the other group members truly “understood what I had been through.”

#### Second-Stage Shelters

Only three of the 40 women (7.5%) accessed second-stage shelters, one of which was identified as Indigenous; offering clients both traditional healing and mainstream individual and group counseling. The women's experiences with the shelter staff and individual counseling varied. One woman described negative interactions with staff members at the Indigenous second-stage shelter. “The organization has power and control. [They] don’t do anything if they don’t like you. I just approached them wrong. I’m one they didn’t like.”

Another woman disclosed that, even though the second-stage shelter had counselors, she continued to work with the counselors at the VAW emergency shelter. “I’d get flashbacks and become all emotional. They were a phone call away.” The other woman commented that the rules at the second-stage shelter were strict; residents were not allowed to work so that they would have the time to focus on their personal healing.

Two women noted the challenges of living in a building with so many other women, including some with addiction issues. Yet, they received support from other residents. “We called ourselves the black boot society. Stick a black boot in the door so it was ajar; that was [a signal]. Women would come by and coffee and hang out; kids would play.”

#### Mainstream Community Counseling Services

In total, 70% (28 of 40) of the respondents accessed support through community counseling services. As mentioned, all five women who identified as LGBTQ or two-spirit accessed community services, with two women accessing LGBTQ-specific services.

The 26 women who accessed generic community counseling agencies commented on what they had hoped to gain, barriers they faced in accessing the services, and their experiences with both group and individual counseling. We could not determine whether the various agencies provided culturally sensitive or culturally appropriate services for Indigenous clients. Further, only one agency clearly had specific knowledge and understanding of IPV.

Six of the 26 women (23.1%) discussed barriers to community counseling. Two women found access difficult because there were not enough counselors, “For every 10 battered persons, there's one counsellor, which makes it very difficult. A lot of [people] give up, like me.” Another woman described the barriers of living in the northern part of the province. For example, after being raped by her partner a woman commented, “It took years to heal because the only therapist that specialized in rape came to our community once a month.”

Twenty-three women discussed their individual counseling. About one-third (8 of 23 or 34.8%) did not find it helpful. Overwhelmingly, these women commented that their counselors were not knowledgeable about IPV and/or lacked the experience to help them. Comments included, “I went through all kinds of counselling: therapists, counsellors, psychologists, psychiatrists. They were never helpful with information,” and “I’ve done almost twenty years of therapy and it wasn’t helping. They had absolutely no idea what I was talking about.”

In contrast, the majority, 18 of the 23 women (78.3%), found the individual counseling helpful, commenting, “I found the best counsellor in the world. He helped me get my power,” *and*, “Talking with her [counsellor] made me stronger. I was able to get my education and eventually leave my relationship because I knew what my husband did to me wasn’t right.”

Seven women described attending IPV-specific treatment groups offered by community agencies. Two women felt pressured by counseling staff to attend groups and declined because they were not comfortable with a group setting. However, five women (19.2%) accessed groups, with three simply stated that they had attended.

*Community LGBTQ Counseling Services*. The two women who had accessed community LGBTQ counseling services had different experiences. One woman had negative experiences with the two agencies she used: one agency offered support, but its fee-for-service was too high. The second woman used a variety of LGBTQ services in both Manitoba and Saskatchewan, all of which she found helpful, describing them as, “Healing … They educated me. They[also] taught me my [Indigenous] culture.”

### Indigenous Healing Practices

Seventeen women (42.5%) saw traditional cultures, teachings, and ceremony as significant in their healing from life experiences and, in particular, from IPV. Two women were raised traditionally; another 15 women learned about traditional values and ceremonies later in their lives.

Thirteen women highlighted the importance of traditional healing/spirituality in their personal lives, commenting, “Being Aboriginal, my spirituality helped get me through. Your roots - it's the core of you. Your faith is what's going to get you through any experience.” And “You have to heal, to find yourself, find your culture. I got to know my culture well; I found myself and how to respect myself.”

These 13 women also commented on the importance of Elders and Healers. “She would tell me things I needed to hear. She was somebody I could trust.” Another spoke of an Elder's comfort. “I would talk about my dream. She would help me interpret or pray for me or burn sweetgrass.” Five women accessed Elders through friends or family, although three of the five mentioned challenges connecting with Elders. “Somebody was going to introduce me to an Elder, but I never heard back.” One woman's natal family was not supportive. “My parents never trusted the spiritual aspect of our culture because my father was a minister. They said it was of the devil.” Another woman noted that, even after finding an Elder, maintaining contact could be difficult. “She got so busy, I just stopped trying.”

Fourteen women described how smudging, prayer, sweats, and circles helped them heal. “We go to ceremony. We get our healing through traditional ways.” Four women mentioned the value of smudging: “We smudge when we feel upset or scared;” and, “I feel more at peace when I’m smudging. It gets rid of the negativity.” Thirteen women described the importance of sweats and circles. “When you keep something inside, it builds up so much emotion. When you let it out, it's not there anymore.” They discussed the significance of having a safe place to share their experiences of IPV. “I go to a sweat lodge and that's where it comes out.”

In summary, the narratives of the 40 women highlighted the importance of accessing VAW shelters, community counseling and traditional culture and healing practices, also identifying barriers and gaps in services.

## Discussion

Although the women had experienced severe IPV, as indicated by the CAS subscales, on the mental health measures they were not generally experiencing severe symptoms of psychological distress, depression, or PTSD, and their QOL was slightly more positive that other studies of abused women using the same measure (Bell et al., 2009; Varcoe et al., 2017). These results are consistent with the entire sample of 658 women participants in the Healing Journey study, about half of whom were Indigenous (Tutty et al., 2020).

Nevertheless, the generally nonclinical mental health scores contrast with studies such as the global systematic review conducted by Chmielowska and Fuhr (2017) that linked Indigenous women's experience of IPV with depression and PTSD, which was further exacerbated by poverty, discrimination, and substance abuse. Heidinger (2021) concluded that 25% of both Indigenous and Non-Indigenous respondents who experienced IPV were dealing with PTSD symptoms. Notably, however, neither study used standardized clinical measures. As well, a proportion of the women in the current study scored in the clinical ranges on depression, PTSD, and psychological distress. Our study results suggest the importance of not assuming that Indigenous women with abusive partners necessarily having mental health issues. Moreover, in a Canadian study of the victimization of Indigenous women, 90% of the respondents self-reported that their mental health was good or very good (Brennan, 2011).

An issue that was unanticipated was that five women identified as lesbian, bisexual, or two-spirit and, thus, two of the abusive partners were female. While recent literature on IPV in the LGBTQ community indicates that IPV rates are higher in the LGBTQ than in the heterosexual community (Decker et al., 2018; Coston, 2021), Ristock et al. (2019) could find no studies focusing on Indigenous Two-Spirit/LGBTQ and IPV. More recently, Heidinger (2021) reported that Indigenous LGBTQ and Two-Spirit people are five times more likely to experience IPV than non-Indigenous LGTBQ.

Only a few women (15%) described having attended residential schools. Nevertheless, the impact of colonization, systemic oppression, and residential schools on generations of their families and communities seems apparent in that 90% of the women had experienced childhood abuse, specifically sexual abuse (67.5%); physical abuse (62.5%); emotional abuse (75%); neglect (45%) and exposure to IPV (70%). The high incidence rate is consistent with Heidinger's Canadian report (2021), concluding that 42% of Indigenous women had experienced physical or sexual abuse “by an adult during childhood compared with 28% of non-Indigenous women” (p. 7; see also Brownridge et al., 2017).

### The Women's Use of Services

It was clear that the 40 Indigenous women were committed to improving their lives through accessing multiple supports, including VAW shelters, second-stage shelters, community counseling agencies, and traditional healing and cultural practices. They often accessed more than one service to assist them in dealing with their partners’ abusive behaviors.

The women's discussion of VAW emergency and second-stage shelters indicates how much the shelters varied, not only in the services offered, but whether the respondents had a choice in participating, and the quality of those services. Negative experiences in VAW and second-stage shelters seemed related to rigid adherence to programming, feeling unheard and/or judged. In contrast, the women's positive experiences were related to feeling respected, heard, and supported through the complexity of their circumstances by staff and other residents.

Congruent with the current study, VAW shelters have consistently identified as an important resource for abused women by providing them and their children with safety and support (Allen et al., 2021; Anderson et al., 2012; Hughes, 2020; Tutty, 2015). In the shelters that offered individual counseling services, over two-fifths of the women reported positive experiences, highlighting the value of being able to reflect and process their experiences of IPV, explore next steps, and learn about IPV and the intergenerational cycle of abuse.

Unfortunately, a small number (18.2%) perceived their counselors to be inexperienced, judgmental, and/or racist, a concern raised by other researchers (Baskin, 2012; Tutty, 2015). A recent article by (Jackson et al., 2015) describes how Blackfoot knowledge and practices were integrated into a rural Alberta VAW shelter, however such initiatives are rare.

There is a considerable body of research on the value of support and therapy groups for women IPV survivors (Allen et al., 2021; Trabold et al., 2018; Tutty, 2017; Tutty et al., 2017), although none are specific to Indigenous women. Notably, support groups are offered in many VAW shelters (Allen et al., 2021; Wathen et al., 2015). Most women (88.9%) who attended support groups in VAW shelters in the current study found them helpful, not only for the counseling or information aspects, but also for the contact and support from the other residents.

In the current analysis, the women's divergent experiences may have been influenced by the staff's general lack of knowledge and understanding of Indigenous cultures. Only one mainstream VAW shelter offered in-house access to elders. Interestingly, Beattie and Hutchins (2015) reported that 63% of shelters stated that they offered culturally sensitive services. In the current study, only one VAW shelter identified itself as Indigenous. Yet, residents of Indigenous VAW shelters may be concerned about their confidentiality when the staff are also members of the community (Jackson et al., 2015; Wuerch et al., 2019).

Only three women accessed second-stage shelters in the current study, which likely reflects how few second-stage shelters are available in the prairie provinces. The women had varied experiences, with one noting issues of power and control from staff, even though it was an Indigenous second-stage shelter.

Funding cuts have reduced the number of second-stage shelters across Canada (Cotter & Burczycka, 2011), further, the existing second-stage shelters tend to be in urban areas (Tutty et al., 2008). Thus, women living on reserve, in rural or northern communities frequently must leave their home communities to access a second-stage shelter. Considering that Indigenous cultures are relational, an Indigenous woman's decision to leave her home community is understandably difficult and could have implications for her well-being (Guggisberg, 2019; Kirmayer et al., 2009; McCormick, 2009).

#### Community Counseling Services

While the Canadian prairie provinces have the highest proportion of Indigenous individuals in the country, the lack of Indigenous-specific counseling services for any issues, but especially for IPV, is striking. Exceptions include a couples-counseling approach to IPV (Riel et al., 2016). Some consider family systems approaches as a better fit with Indigenous couples and families (Baskin, 2012; Kirmayer et al., 2009; McCormick, 2009). Notably, Indigenous-specific approaches to IPV are rare. Further, although a number of women in the current study had been sexually assaulted by partners, none mentioned using sexual assault services, congruent with Du Mont et al. (2017).

A paucity of literature has examined abused Indigenous women's use of community counseling, yet 70% of the women in the current study accessed such counseling. It was not possible to determine whether the counselors had knowledge of either IPV or Indigenous cultures. Nevertheless, over three-quarters of the women (78.3%) found the counseling empowering because it gave them a place to process, reflect, and gain strength, which is consistent with the literature (Anderson et al., 2012). The most common concerns were difficulty accessing services, fees for service, limited number of sessions, and/or feeling pressured to attend groups.

Interestingly, the women who identified as LGBTQ or two-spirit accessed only mainstream counseling services; none went to VAW shelters (Ristock et al., 2019), perhaps fearing prejudice and oppression from VAW shelter staff because of their gender identity. The two women who accessed community LGBTQ-specific counseling services had different experiences. One found her confidentiality breached, while the other one found it helpful, describing how it taught her about Indigenous cultures and healing practices.

In summary, the women generally indicated that the counseling and support they received from services was helpful. The women's positive experiences seemed associated with feeling respected, heard, and supported by staff. Nevertheless, it was not clear whether the counselors had any training/understanding of Indigenous history or culture, nor whether community counselors had any training/understanding of IPV.

### Indigenous Healing and Cultural Practices

Notably, as mentioned, only two women were raised in traditional Indigenous families; the other 15 connected with Indigenous healing practices later in life. All 17 spoke of the importance of traditional culture, teachings, and ceremony in their healing from IPV. They discussed how practices such as attending to dreams and visions, participating in circles, using Indigenous ceremonies such as smudging, sweats, and Sun Dance strengthened their relationships with the natural and spiritual world. These practices not only enhanced their healing journeys but also aligned with existing literature (Bird, 2007; Baskin, 2012; Kirmayer et al., 2009; McCormick, 2009; Olsen Harper, 2006). Seven women spoke of the importance of accessing Elders, yet three women had difficulty accessing and maintaining contact with them.

### Implications for Counselors

In the current study, 70% of the women accessed community counseling services, highlighting the need for counselors and students to have courses about IPV, in Indigenous, heterosexual and LGBTQ communities. Western counseling paradigms stress the need for evidence-based practice (Gilgun, 2005) and Indigenous healing approaches are generally not evaluated (Bird, 2007; Gone, 2009; Jackson et al., 2015; Lucero, 2011). Indigenous healing strategies are assessed more by qualitative, word-of-mouth endorsements. (Gone, 2009).

As indicated, the respondents experienced traumatic events in response to their partner's abusive behaviors, child maltreatment and intergenerational trauma. Yet the women's average scores on the PTSD checklist were not in the clinical range that would suggest a PTSD diagnosis. Nevertheless, some women were in the PTSD diagnostic range and others were dealing with trauma symptoms. Both groups could benefit from trauma-informed counseling that addresses women's mental health needs in context and considers community, social and societal factors (Mango et al., 2019; Sullivan, 2018). Providing courses on trauma-informed care as part of counselor education seems crucial.

Some authors suggest that Indigenous healing approaches may work well in concert with mainstream interventions (Bird, 2007; Brave Heart et al., 2011; Jackson et al., 2015). Others argue that Indigenous healing approaches should be considered as interventions in and of themselves. not simply “complementary” activities (Gone, 2009; Lucero, 2011). This suggestion merits additional consideration by counselors. Finally, while some Indigenous women embrace traditional healing, others use mainstream counseling and traditional healing practices in concert, and others only use mainstream interventions. Women need to be aware of both traditional and mainstream approaches so they may choose what interventions and approaches fit best.

### Limitations and Strengths of the Current Study

A central concern in qualitative secondary analysis is that limitations from the primary study also apply to subsequent analyses (Long-Sutehall et al., 2011). Despite the training on conducting semi-structured qualitative interviews, some research assistants treated the interview schedule and probes as though they were a structured questionnaire. Further, in 21 of the 40 interviews, the interviewers did not ask the suggested probe about whether being Indigenous had influenced their experiences with various services. Notably, though, even with inexperienced interviewers, the women were persistent in discussing the severity of their partner's abuse; placing their partner's abusive behavior in context by sharing the violence they had experienced throughout their lives, as well as the oppression and racism from partners and/or the dominant society. The women were recruited from IPV-specific agencies, which biases the sample towards those open to accessing assistance. As such, the results cannot be generalized to Indigenous abused women in Canada's prairies.

Data from the primary society was collected between 2007 to 2009, which could lead some to question the relevance of the women's experiences to current day circumstances. However, a comparison of the Burczycka and Cotter's national survey of shelters for abused women in Canada (2010) to the most recent survey by Beattie and Hutchins in 2015, seem not to indicate any significant changes.

Using measures that examine potential issues as well as the women's strengths highlights the complexity of Indigenous women's responses to IPV. Moreover, little previous literature has addressed Indigenous women's use of either mainstream counseling agencies or Indigenous healing practices.

## Conclusion

There is a paucity of literature on the experiences of Canadian Indigenous women abused by intimate partners. This study helps address this gap while highlighting the severity of the IPV, including intimate partner sexual abuse against the respondents. It describes their use of VAW emergency and second-stage shelters, mainstream community counseling, traditional healing, and cultural experiences to assist them in their healing journeys. Their resilience was notable, as is evidenced by their average nonclinical scores on mental health measures (Ogden & Tutty, 2024). Even though the women faced numerous challenges, in general, they maintained their strengths and mental health.

The women shared many intimate details of their lives, including sensitive information about their relationships with family and abusive partners. It was important that their narratives are heard and respected. It is only by honoring the voices of the respondents that we can begin creating practices, programs and services that are truly useful. Taking such steps can help us move towards creating a more equitable, decolonized society.
